# Clinical, Genomic, and Transcriptomic Characteristics of Patients with Metastatic Renal Cell Carcinoma Who Developed Thromboembolic Events

**DOI:** 10.15586/jkcvhl.v11i3.319

**Published:** 2024-07-31

**Authors:** Gliceida Galarza Fortuna, Beverly Chigarira, Vinay Mathew Thomas, Kamal Kant Sahu, Shruti Adidam Kumar, Nishita Tripathi, Nicolas Sayegh, Neeraj Agarwal, Umang Swami, Benjamin L. Maughan, Haoran Li

**Affiliations:** 1 Division of Medical Oncology, Department of Internal Medicine, Huntsman Cancer Institute, University of Utah, Salt Lake City, UT, USA;; 2 Division of Medical Oncology, Department of Internal Medicine, The University of Kansas Hospital, KC, KS, USA

**Keywords:** cancer, kidney, renal cell carcinoma, thromboembolic events

## Abstract

Thromboembolic events (TE) are a common complication in patients with metastatic renal cell carcinoma (mRCC) and are associated with poorer clinical outcomes. However, the incidence of TE and clinical and genomic characteristics of patients with mRCC who develop this complication are poorly understood. Herein, we describe the incidence and clinical features of patients with mRCC with or without TE at our institution, and examine their association with the underlying genomic and transcriptomic characteristics of the tumor. This retrospective study included all consecutive cases of mRCC seen at our institution. A CLIA-certified lab performed tumor genomics and transcriptomics. Patients were classified based on the presence of a TE within the first year of diagnosis. Three hundred and seventy patients with mRCC were included in the study. TE was seen in 11% (42) of the patients. Patients with favorable International mRCC Database Consortium (IMDC) risk were less likely to develop a TE. In contrast, patients receiving combination treatment with a tyrosine kinase inhibitor (TKI) and an immune checkpoint inhibitor were more likely to develop a TE. No difference in overall survival among patients with or without TE was observed (52 vs. 55 months; HR 0.85, 95% CI 0.5574–1.293, p = 0.24). The most upregulated pathways in mRCC with TEs versus those without were the xenobiotic metabolism and mTORC1 signaling pathways. Our findings suggest potential biomarkers that, after external validation, could be used to better select patients who would benefit from prophylactic anticoagulation.

## Introduction

Neoplastic conditions have historically been associated with the development of thrombotic complications, often presenting as the initial signs of cancer (1). About 20% of patients who present with their first thromboembolic event (TE) have an underlying cancer diagnosis (2,3). Furthermore, thromboembolic events can also lead to significant morbidity and mortality in cancer patients. It has been reported that 14% of in-hospital mortality in cancer patients is due to complications from a pulmonary embolism (4).

Renal cell carcinoma is the most common kidney cancer, accounting for 85% of all malignant kidney neoplasms (5). In patients with metastatic kidney cancer, the overall 2-year incidence of TE has been estimated to be approximately 6.0 per 100 patient years (6).

Khorana et al. conducted a retrospective study on patients with metastatic renal cell carcinoma (mRCC) treated with immunotherapy between January 2015 and December 2019. Their analysis found that 11% of patients had a documented TE from the diagnosis of mRCC to the time of immunotherapy initiation (7). The study did not find any association between clinical variables and the development of thromboembolic complications, but it did observe worse outcomes in patients who developed a thrombotic complication.

Given that scores based on clinical characteristics are an inadequate tool for predicting the risk of TE in patients with cancer, recent studies have investigated the role of molecular aberrations and their association with TE (8). For example, changes in genes such as KRAS,

ALK, ROS 1, and IDH1 have been associated with an increased risk of TE (9). However, to date, no studies have yet examined the impact of molecular changes on TE in patients with RCC.

Herein, we aim to assess the clinical, tumor genomics, and tumor transcriptomic characteristics of patients with mRCC who develop clinically relevant TE and compare them with patients with mRCC with no thromboembolic complications.

## Materials and Methods

### Patients selection

We performed a single-center retrospective cohort study of consecutive patients diagnosed with mRCC between August 2000 and January 2023 at the University of Utah/Huntsman Cancer Institute. All patients with histologically proven mRCC were included in the analysis. Patients with tumor thrombus (TT) only detected on post-nephrectomy pathology evaluation, with no clinical or imaging correlation, were excluded.

The medical records were reviewed to obtain patients’ demographics, baseline, and genomic characteristics. Venous thromboembolisms (VTEs) were divided into deep venous thrombosis (DVT), pulmonary embolism (PE), and tumor thrombus (TT), while arterial thromboembolic events (ATEs) were divided into thromboembolic cerebral vascular events (TE-CVA) and arterial thrombosis (AT). Only TE diagnosed on clinical images (US, CT, MRI, V/Q scan) were included. This research study received approval from the University of Utah Institutional Review Board (IRB).

### Genomic and transcriptomic profiling

Comprehensive genomic profiling was performed from DNA extracted from formalin-fixed, paraffin-embedded tumor tissue samples by a CLIA-certified next-generation sequencing panel (Tempus Labs, Inc or Caris Life Sciences). RNA sequencing was performed on tumor tissue samples using either the Tempus xT assay or the Caris Molecular Intelligence® platform. The Tempus xT assay is a targeted RNA sequencing panel that covers over 1400 genes involved in cancer biology, including key oncogenic drivers and immune markers. The Caris Molecular Intelligence® platform is a comprehensive tumor profiling platform that includes RNA sequencing.

### Statistics

The clinical and genomic data were analyzed utilizing descriptive statistical analysis using median with 95% confidence interval (CI) and percentage (number of events). Furthermore, we calculated Pearson Chi-square, Fisher’s exact test, or T-test to obtain p-values to compare the frequency or mean between patients with and without TE events to determine whether significant variation existed within these two groups. Simple survival analysis utilizing the Kaplan-Meier methods was performed to estimate and compare overall survival in patients with and without TEs. All statistical analysis was done on GraphPad Prism. A p-value of <0.05 was considered statistically significant.

### Transcriptomic analysis

Differential gene expression analysis was performed using DeSeq2 in Bioconductor software to identify differentially expressed genes between the two cohorts. The DeSeq2 results included the Log2 Fold change, Wald-Test p-values, and Benjamini-Hochberg adjusted p-values for each differentially expressed gene. A fold change of an absolute of 2 and an adjusted p-value of less than 0.05 were used as the criteria for differential expression.

Gene set enrichment analysis (GSEA) was performed to identify enrichment. Positive normalized enrichment scores determined upregulated pathways, while negative enrichment scores determined down-regulated pathways in the TE cohort. GSEA is a widely used bioinformatics tool that assesses whether a set of genes is enriched in a predefined pathway or biological process. The gene sets used in this analysis were obtained from the Molecular Signatures Database (MSigDB) (10,11). All bioinformatic analysis was conducted in R-Studio, version 4.1.1.

## Results

A total of 370 consecutive patients with mRCC were included. Of these, 42 (11%) developed a thromboembolic event (TE) within 1 year of diagnosis. The median age of patients who developed a TE was 64 [95% confidence interval (CI) 60–66] and 61 (95% CI 60–63) years for those with no documented thromboembolic complication (p-value = 0.1883). Eighty-three percent of patients with a documented TE were male, while 68% of those who did not were male (p-value = 0.0503). The majority of patients in both cohorts were white (88% vs. 90%, p-value = 0.5865), had pure clear-cell histology (90% vs. 78 %, p-value = 0.0691), and a Fuhrman grade >2 (76% vs. 76%, p-value > 0.999). Patients with International mRCC Database Consortium (IMDC) favorable risk were less likely to develop a TE (0% vs. 14%, p-value = 0.01). Furthermore, both cohorts were similar in obesity rate (BMI > 30) and nephrectomy status. Table 1 presents all clinical characteristics of mRCC patients with and without TE. The most common site of metastatic involvement in both cohorts was the lung; there was no difference in the rate of TE according to the metastatic site (Table 2).

**Table 1:** Clinical characteristics of patients with mRCC with and without thromboembolic complications.

Characteristics, n (%)	TE (N=42)	No TE (N=328)	*p*-value
Age, median (95% CI), y	64 (60–66)	61.5 (60–63)	0.19
Male	35 (83)	225 (68)	0.05
BMI, median (95% CI)	30.44 (27.50–34.15)	27.99 (27.01–28.86)	0.09
Race			
White	35 (88)	285 (90)	0.59
Black/African American	0	2 (0.6)	
Hispanic/Latino	4 (10)	18 (5.6)	
Asian/Pacific Islander	1 (2)	9 (2.8)	
Native American	0	3 (1)	
Nephrectomy	34 (83)	267 (81)	1.00
Fuhrman Grade			
<=2	8 (24)	58 (24)	1.00
>2	25 (76)	182 (76)	
clear cell RCC	30 (90)	258 (78)	0.07
IMDC risk classification			
Favorable	0	43 (14)	*0.01*
Intermediate	24 (73)	174 (59)	0.74
Poor	9 (27)	80 (27)	0.85
Synchronous metastasis	13 (31)	140 (43)	0.15
Karnofsky PS <80	6 (17)	53 (17)	1.00
Chronic kidney disease	8 (23)	84 (28)	0.69
Anemia	25 (68)	188 (61)	0.48
Neutrophilia	6 (17)	62 (18)	0.82
Hypercalcemia	4 (11)	20 (7)	0.31
Thrombocytosis	5 (14)	55 (18)	0.65

**Table 2:** Distribution of metastatic involvement in patients with mRCC with or without thromboembolic complications.

Site of metastatic involvement n (%)	TE (N=42)	No TE (N=328)	*p*-value
Lymph node	14 (33)	155 (47)	0.09
Lung	27 (64)	221 (67)	0.69
Brain	6 (14)	28 (8)	0.23
Liver	6 (14)	59 (18)	0.55
Bone	14 (33)	100 (30)	0.71
Adrenal	3 (7)	48 (15)	0.18
Pancreatic	5 (12)	19 (6)	0.13
Other	6 (14)	93 (28)	0.05

**Figure 1:**
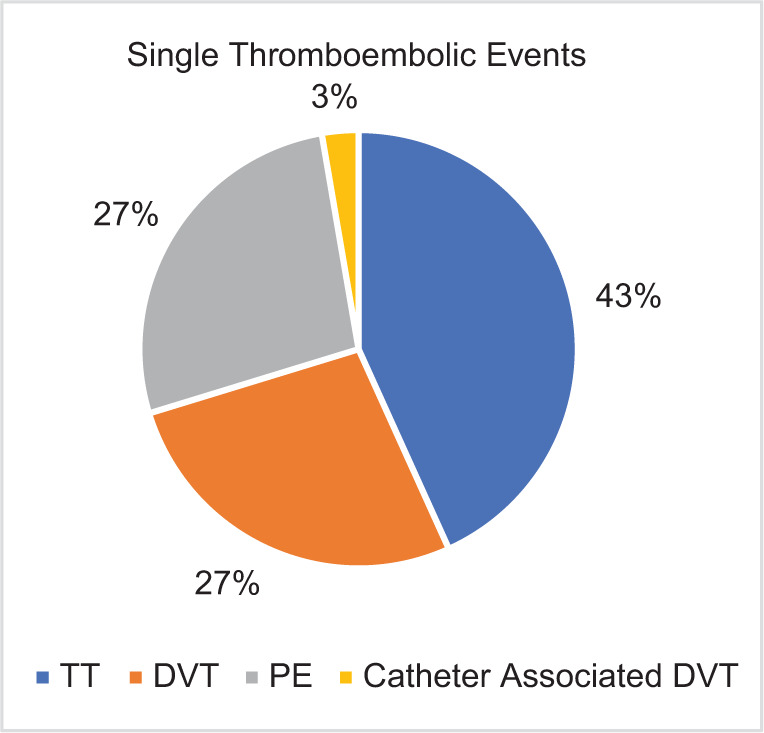
Rates of thromboembolic events in patients with mRCC.

In patients who developed a TE, a venous event was the most commonly observed in 41 (98%) of the patients within the cohort; ATE was seen in two patients, one who developed a thromboembolic CVA, and another who developed an arterial thrombus leading to a splenic infarct and hepatic infarct. The patient who had developed a hepatic and splenic arterial thrombus also had a documented VTE. Of the patients with confirmed VTE, 37 (90%) had a single event, while four (10%) had multiple TE recorded. Of the patients with a single documented event, TT was the most common event seen in 16 (43%) of the patients; 10 (27%) had a DVT, 10 (27%) had a PE, and 1 (3%) had a catheter-associated DVT (Figure 1). The median time to event was 2 (range 0–11) months from the time of metastatic diagnosis, with 60% of the events occurring within the first 3 months following diagnosis of metastatic disease. In 15 (35%) of the patients, the diagnosis of a TE preceded the diagnosis of mRCC by a median of 103 (range 2–397) days. A total of 35 patients (83%) started anticoagulation following documentation of a thromboembolic event. The most commonly utilized pharmacologic anticoagulation (AC) was a direct-acting oral anticoagulant used in 24 patients (69%), followed by warfarin (17%) and low-molecular-weight heparin (14%). The median time of anticoagulation duration was 748 days (range 44–3432 days).

**Figure 2:**
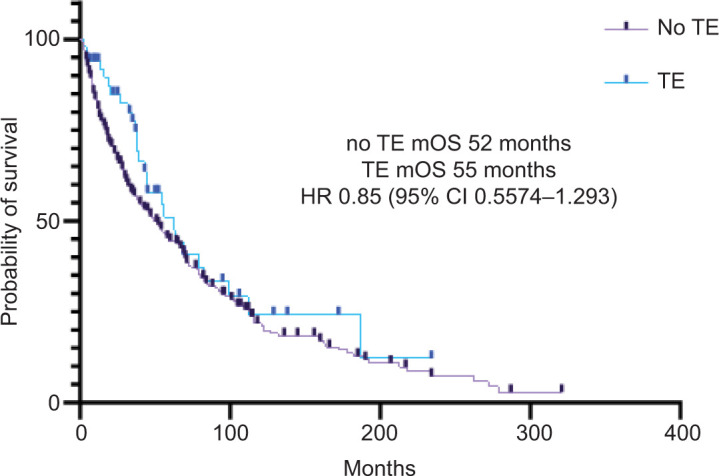
Overall survival of patients with mRCC with and without thromboembolic complications.

Forty-eight percent of patients with documented TE developed the event prior to initiating first-line treatment. The median time from TE to treatment initiation was 4 months (range 0–33 months). Similarly, 40% of patients developed TE after initiating first-line treatment; the median time from treatment initiation to TE was 2 months (range 0–90). Twelve percent of patients who developed a TE never started treatment for their mRCC. Of patients who initiated therapy prior to developing a TE, those who received monotherapy with a tyrosine kinase inhibitor (TKI) were less likely to develop a TE (p=0.0009). However, patients on combination therapy with an immune checkpoint inhibitor (ICI) and a TKI were more likely to develop a TE (p=0.0010) (Table 3). The median overall survival (mOS) in patients without and with TE was not statistically significant, 52 versus 55 months, respectively (HR 0.85, 95% CI 0.5574–1.293, p-value = 0.24) (Figure 2).

**Table 3:** Treatment characteristics of patients with mRCC who did or did not develop thromboembolic complications during treatment.

Treatment n (%)	TE (N=17)	Non-TE (N=317)	*p*-value
ICI only	3 (18)	34 (11)	0.42
TKI only	5 (29)	222 (70)	0.0009
TKI-ICI	6 (35)	24 (7)	0.0019
Other	3 (18)	37 (12)	0.41

Of all patients with documented TE, 21 (50%) had available genomic data, while 126 (38%) of those without documented TE had this information available. As expected, the most commonly seen somatic genomic alteration in both groups was VHL (76% vs. 58%), followed by PBRM1 (57% vs. 41%), TP53 (28% vs. 23%), MTOR (19% vs. 10%), and SETD2 (14% vs. 17%). The frequency of these genomic alterations was well-balanced between the two groups (Table 4).

**Table 4:** Genomic alterations present in patients with mRCC with and without thromboembolic complications.

Gene, % (n)	VTE (N=21)	No VTE (N=126)	*p*-value
VHL	76 (16)	58 (74)	0.15
PBRM1	57 (12)	41 (52)	0.23
SETD2	14 (3)	17 (22)	1.00
BAP1	9 (2)	11 (14)	1.00
TP53	28 (6)	23 (29)	0.59
TERT	0	10 (12)	0.22
CDKN2A	5 (1)	14 (15)	0.47
KDM5C	9 (2)	11 (14)	1.00
PTEN	5 (1)	10 (13)	0.69
ARID1A	9 (2)	11 (14)	1.00
MTOR	19 (4)	10 (13)	0.27

We identified 29 patients with available RNA-seq data for transcriptome analysis, of which four patients had a TE. GSEA revealed the following five pathways to be the most upregulated in patients with TE compared to those without a documented TE: xenobiotic metabolism, mTORC1 signaling, oxidative phosphorylation, reactive oxygen species, and adipogenesis. Furthermore, the following five pathways were the most downregulated in TE versus no TE mRCC patients: UV response, interferon-gamma, interferon alfa, TFG beta, and WNT Beta-catenin (Table 5). Using GSEA, we identified the most upregulated genes in each pathway, as seen in Table 6.

**Table 5:** Differential gene set expression analysis scores in mRCC with TE versus without TE.

Pathway	Normalized enrichment score	*p*-value	q-value
Xenobiotic metabolism	2.08	1.77E-11	<0.0001
mTORC1 signaling	1.99	1.14E-09	<0.0001
Oxidative phosphorylation	1.98	1.97E-09	<0.0001
Reactive oxygen species pathway	1.92	4.58E-05	<0.0001
Adipogenesis	1.856	5.75E-07	<0.0001
Glycolysis	1.77	2.81E-06	<0.0001
E2F targets	1.66	7.59E-05	<0.0001
Myc targets V2	1.65	0.003	0.004
Fatty acid metabolism	1.63	3.34E-04	<0.0001
Estrogen response late	1.63	1.61E-04	<0.0001
Unfolded protein response	1.62	0.001	0.002
Estrogen response early	1.62	1.77E-04	<0.0001
Bile acid metabolism	1.62	0.001	0.002
UV response up	1.51	0.003	0.004
Peroxisome	1.48	0.01	0.011
G2M checkpoint	1.48	0.003	0.004
Cholesterol homeostasis	1.45	0.02	0.023
Heme metabolism	1.36	0.02	0.020
Myc targets V1	1.36	0.02	0.020
UV response down	–1.43	0.005	0.006
Gamma response	–1.43	0.002	0.002
Interferon alpha response	–1.63	8.57E-04	0.002
TGF-beta signaling	–1.69	0.002	0.003
Wnt/Beta-Catenin signaling	–1.82	0.001	0.002

**Table 6:** Differential gene expression showing the 5 most expressed genes in each pathway.

Pathway	Highly expressed genes
Xenobiotic metabolism	*HSD17B2, AKR1C2, PC, FBLN1, CYP2S1*
mTORc1 signaling	*ELOVL6, PSPH, DHCR24, SLC7A5, HMGCS1*
Oxidative phosphorylation	*POR, ATP5PB, OGDH, ATP5F1C, DECR1*
Reactive oxygen species pathway	*SOD2, GSR, ATOX1, GPX3, FTL*
Adipogenesis	*ELOVL6, C3, CMPK1, ITIH5, APOE*
Glycolysis	*PC, TFF3, GMPPA, CITED2, HOMER1*
E2F targets	*GSPT1, SPC24, POLD1, CDCA8, TIMELESS*
Myc targets V2	*RRP12, NOP16, AIMP2, SLC19A1, SORD*
Fatty acid metabolism	*EPHX1, DHCR24, PCBD1, ACAT2, GPD1*
Estrogen response late	*PRLR, TPD52L1, S100A9, TFF3, DNAJC12*
Unfolded protein response	*SLC7A5, ATP6V0D1, TUBB2A, BAG3, ERN1*
Estrogen response early	*TPD52L1, TSKU, RHOBTB3, SLC7A2, MINDY1*
Bile acid metabolism	*GC, HSD3B7, DHCR24, IDH2, ABCD1*
UV response up	*SOD2, CLTB, EPHX1, BAK1, PPIF*
Peroxisome	*SOD2, HSD3B7, DHCR24, IDH2, ABCD1*
G2M checkpoint	*SLC7A5, CHAF1A, E2F3, GSPT1, SLC7A1*
Cholesterol homeostasis	*ACAT2, HMGCS1, CLU, ETHE1, DHCR7*
Heme metabolism	*C3, PC, IGSF3, SLC11A2, RANBP10*
Myc targets V1	*NOP16, PSMC4, SERBP1, TUFM, ILF2*
UV response down	*TGFBR3, RGS4, PIK3CD, VAV2, FHL2*
Gamma response	*TOR1B, DDX58, NFKB1, IL4R, IL2RB*
Interferon alpha response	*PARP9, ADAR, LPAR6, LAMP3, PARP14*
TGF-beta signaling	*LTBP2, SMURF1, FURIN, RHOA, WWTR1*
Wnt/Beta-Catenin signaling	*KAT2A, WNT6, ADAM17, NCSTN, TCF7*

## Discussion

Thromboembolic complications are commonly seen in patients with underlying malignancies. As such, the risk of developing venous thromboembolism in patients with cancer is approximately 4%–20% higher compared to those with no history of cancer, while the risk of arterial thrombosis is 2%–5% higher (12). However, not all cancers carry the same risk of thrombosis (13). In patients with renal cell carcinoma, the incidence of TE has been estimated to range from 8.3% to 12% (7,14). Most of these events are VTEs, with arterial events occurring in approximately 2% of patients (15). The pathophysiology behind this hypercoagulable state associated with cancer is believed to be the secretion of circulating P-selecting and mucins from the primary tumor, leading to the formation of microthrombi and the release of tissue-factor rich vesicles which ultimately lead to platelet aggregation and cytokines production which disrupts the endothelium (16).

Our results confirm the previously described frequency of thromboembolic events in patients with mRCC, with a prevalence of 11%. Similarly, most of these events were venous in etiology, with arterial events representing only 5% of all the described thrombotic events. Furthermore, most of these events (60%) occurred within 3 months of diagnosis, which is consistent with previously reported data (14).

In our cohort, patients with documented TE were less likely to have IMDC favorable risk disease (0% vs. 14%, p= 0.0082), while there was no difference in the frequency of intermediate and poor risk disease in patients with and without documented events. This differs from recently reported results, in which a difference in the incidence of TE was not seen based on the IMDC risk group (17). However, it is to be noted that major registration trials in metastatic kidney cancer exclude patients with a history of thrombosis 6 months prior to randomization, and hence the patient population enrolled in the clinical trials may not provide the true prevalence of TE associated with mRCC. Although our study did not assess the association between the Khorana score and thromboembolic complications in patients with mRCC, previous studies have shown that the Khorana score does not accurately predict the development of thromboembolic complications in patients with mRCC (18). This information, once validated, could be practice-informing for physicians by warranting initiating prophylactic anticoagulation on patients with IMDC intermediate and poor risk mRCC regardless of their Khorana score.

Recent research studies have suggested that ICIs are associated with an increased risk of venous and arterial thromboembolic events (19,20). This increased risk of thromboembolic complications with ICI has also been documented in patients with mRCC (15,17). Patients who received ICI therapy in our cohort were more likely to develop a TE (18% vs. 11%). Although this difference did not reach statistical significance (p=0.41), this could be because of our small sample size. Similarly, patients receiving combination therapy with a TKI/ICI-based regimen were more likely to develop a TE (p = 0.0019), which has been previously described (17,21). However, in clinical trials this increased risk of TE has not been described. For example, in the phase 3 CheckMate 9ER trial, comparing nivolumab plus cabozantinib versus sunitinib in the first-line treatment of mRCC; the rate of venous thromboembolic events was less than 1% (22). Furthermore, the prevalence of TE with other ICI and/or TKI-containing regimens was not described (23–29). As mentioned above, the lower rates of TE events reported in clinical trials can be explained by the exclusion of patients with a history of VTE or PE within 6 months of enrollment in mRCC clinical trials. These data suggest a need for more extensive studies to understand the risk of TE complications in real-world mRCC patients who received immune checkpoint and TKI-based combination therapies since these are the current cornerstone of treatment for mRCC. The results of these studies in real-world patients could serve as a benchmark for the development of a tool to predict the risk of TE in patients with mRCC. It is important to mention that the cohort of patients analyzed for this study were diagnosed between August 2000 and January 2023. ICI-based combination therapies were first approved by the FDA for the treatment of mRCC in April 2018. Of the patients included in this study, only 75 patients (22%) were treated after April 16, 2018, which explains the high proportion of patients receiving TKI only in the first-line setting. As such, future studies should assess the risk of TE in patients treated with ICI-combination regimens, as these are the preferred current regimens for this patient population.

Lastly, we evaluated the tumor gene expression profile in mRCC patients with and without TE. Although limited by the sample size, our observations remain thought-provoking and deserving of future validation. The enzymes associated with xenobiotic metabolism are involved in two major biological processes: activating substrates into more reactive metabolites through major cytochrome P450 enzymes or clearing out metabolic active substances (30). This pathway has been associated with the development of RCC, and acquiring resistance to treatment (31,32). This was the most upregulated pathway noted in mRCC patients with documented TE compared to those without. The specific cytochrome P gene overexpressed in the xenobiotic metabolism pathway was CYP2S1. Thum et al. showed that CYP2S1 is involved in inflammation by modulating the inflammatory process through the metabolism of cyclooxygenases and lipoxygenases (33). The presence of CYP2S1 in tissue affected by inflammation supports its pro-inflammatory role (34). In RCC, CYP2S1 overexpression has been associated with worse survival (35). Thus, we hypothesize that the upregulation of the xenobiotic metabolism pathway and overexpression of the CYP2S1 gene is associated with an aggressive and pro-inflammatory biology that offers appropriate lieu for thromboembolic events in mRCC patients.

The mTORC1 pathway was the second most upregulated pathway in mRCC patients with TE versus no TE. mTORC1 functions include regulation of cell growth and metabolism (36). This pathway has also been involved in the functions of endothelial cells, which are essential for angiogenesis and endothelial proliferation (37). In RCC, activation of the mTOR pathway, downstream of the PI3-K/AKT pathway, leads to carcinogenesis by directly promoting the growth of tumor cells or by increasing the expression of hypoxia-inducible factor (HIF)-1α and HIF-2α, which are also involved in renal tumorigenesis (38,39). Although with limited efficacy, mTOR inhibitors have been used to treat patients with RCC (37). Interestingly, activation of mTORC1 has been shown to be associated with venous thrombosis (40,41). Our findings suggest that there might be an association between mTORC1 upregulation and the risk of TE in patients with mRCC. This could also serve as a predictive biomarker for thrombotic complications in mRCC patients.

This study, while offering valuable insights into thromboembolic complications in patients with mRCC, is not without its limitations. First, our sample size was relatively small, which might have affected the statistical power to detect certain differences, particularly concerning the risk associated with ICI monotherapy. Additionally, our observational design means that causality cannot be established between the observed associations, only correlations. While we observed interesting gene expression profiles related to thromboembolic complications, these findings are based on a limited number of patients and hence require further validation in a larger cohort. Moreover, potential confounders, which were not accounted for, might have influenced some of our observations. Finally, the generalizability of our results might be limited due to potential selection bias or the specific demographic and clinical characteristics of our study population. Future studies with a more extensive sample, diverse patient population, and perhaps a prospective design would be beneficial in addressing some of these concerns.

## Conclusions

Our data suggest that patients with mRCC have an increased risk of developing TE, with VTE being the most frequently seen event. Furthermore, in our cohort, patients with intermediate and poor IMDC risk were more likely to develop a TE than patients with favorable IMDC risk. Treatment with ICI-TKI combination regimens was also associated with an increased risk of TE. Although there was no association between genomic alterations and risk of TE, we observed an upregulation of the xenobiotic metabolism pathway leading to overexpression of the CYP2S1 gene and upregulation of the mTORC1 pathways in patients with TE compared to those without. These data suggest the need to further explore the role of the CYP2S1 gene in RCC and inflammation in order to better characterize patients at risk of developing TE. The results of our study, along with the multiple previously published data, suggest a potential role for prophylactic anticoagulation in patients with mRCC, especially those with an intermediate or poor IMDC risk undergoing treatment with ICI-TKI regimens.
